# An open-source underwater robotics platform for aquatic research & exploration

**DOI:** 10.1016/j.ohx.2025.e00715

**Published:** 2025-11-17

**Authors:** Scott Mayberry, Jinzhi Cai, Ruochu Yang, Junkai Wang, Fumin Zhang

**Affiliations:** Georgia Institute of Technology, Hong Kong University of Science and Technology, United States

**Keywords:** Autonomous underwater vehicle, Open-source robotics, Underwater sensing, Marine exploration, Environmental monitoring

## Abstract

Underwater research is often constrained by the specialized expertise, high costs, and dedicated facilities required for traditional aquatic experiments, limiting participation from smaller research groups. In response, we introduce an open-source miniature underwater robot (MUR) platform that broadens access to aquatic research and exploration while simplifying experimental implementation. Built on a fully networked ROS ecosystem, our platform integrates advanced sensing, control, and versatile communication capabilities to support real-time data acquisition and sensor fusion. Its modular 5-degree-of-freedom propulsion system enables precise position, yaw, and roll control, with passive pitch stability through neutral buoyancy supporting steady station-keeping and dynamic trajectory tracking. Equipped with multiple cameras, the system facilitates advanced perception tasks essential for complex underwater operations. Moreover, WiFi, radio, and high-speed Ethernet tethering ensure communication in shallow water environments, enabling both autonomous operation and tethered deployments. This open, modular architecture reduces barriers to underwater research while promoting collaborative innovation and establishing a shared research infrastructure for marine science and robotics.

## Graphical abstract

**Figure:**
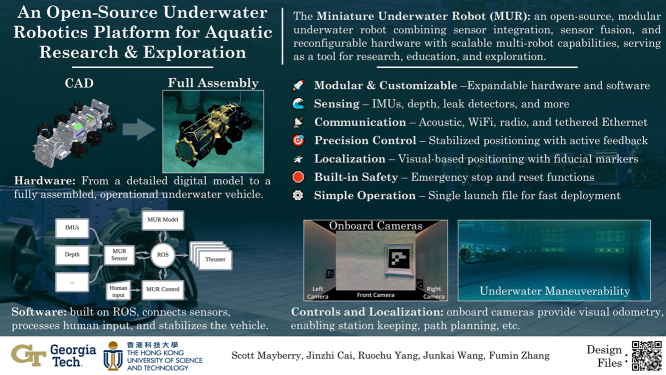

## Specifications table

**Table:** 

Hardware name	Miniature Underwater Robot (MUR)
Subject area	Environmental, planetary and agricultural sciences
Hardware type	Field measurements and sensors
Closest commercial analog	BlueROV2

Open source license	•MIT License (software) • CERN Open Hardware Licence Version 2 - Permissive (hardware) Combined License File: https://doi.org/10.5281/zenodo.14968240, Path: LICENSE.md

Cost of hardware	$850–$2000 (cost options in Table 4, depends on self-3D print/commercial 3D print and low/high-end thrusters)

Source file repository	Zenodo: https://doi.org/10.5281/zenodo.14968240 Github: https://github.com/scottmayberry/MUR

## 1 Hardware in context

Tethered (remotely operated vehicles, ROVs) and untethered (autonomous underwater vehicles, AUVs) systems have become essential platforms for data collection in oceans, lakes, and estuaries [1], [2], [3]. These underwater robots have diversified into a broad spectrum of platforms to perform specialized research tasks, with robots ranging from low-cost, open-source systems to highly specialized, industrial-grade machines. This expansion has helped to enable the next aquatic research frontier involving fleets of aquatic robots performing distributed sampling in aquatic environments [4], including (1) tracking marine life to understand the life cycles of sharks, jellyfish, lobsters, etc. [5]; (2) monitoring and tracking fast-evolving plumes, algae, or other dynamic features [6]; and (3) search and rescue or other dense sampling missions [7], [8].

Commercially available platforms such as the BlueROV2 have set a benchmark in affordability and performance. Built with a six-thruster vectored configuration, the BlueROV2 supports a wide range of inspection and research tasks [9]. In contrast, the Blueye Robotics X3 Underwater delivers state-of-the-art features including 4K imaging and a depth capacity of up to 305 m, though its price tag of nearly $30,000 limits its deployment primarily to well-funded research initiatives [10].

For specialized applications, systems like those used by SARbot UK exemplify the use of advanced, remote-controlled underwater robots for search and rescue missions, with unit costs reaching up to £140,000 [10]. On an even larger scale, industrial platforms such as the Ultra Trencher 1 — designed for trenching submarine pipelines — are valued at roughly £10 million and highlight the extreme end of underwater robotics tailored for deep-water industrial applications [11].

The growth of commercially available underwater vehicles and advances in aquatic research have attracted significant interest from the computing and engineering communities. However, effective cross-disciplinary collaboration is hindered by a lack of shared infrastructure. Underwater robotics requires specialized facilities, expert personnel, and considerable financial resources—resources often out of reach for smaller research groups. Despite recent cost reductions for such platforms, these systems remain expensive for many investigators, and the absence of open-access infrastructure further limits participation. These challenges are underscored in the November 2018 report by the National Science & Technology Council [12], which highlights shared research infrastructure as a top priority for advancing ocean science and technology.

Positioned within this underwater vehicle spectrum, our proposed Miniature Underwater Robot (MUR) bridges the gap between high-end, proprietary systems and the growing need for accessible, community-oriented research tools. Priced between $850 and $2000, the MUR is a modular, open-source platform that lowers both financial and technical barriers to underwater exploration. Its ROS-based software stack supports real-time data exchange and rapid integration of new sensors and control algorithms, enabling researchers to customize the platform for diverse applications — from marine ecology surveys and dynamic plume tracking to search and rescue operations — making it especially well-suited for shared research environments.

The significance of the MUR is further highlighted by its integration into community-driven initiatives like the μNet project [13]. The μNet infrastructure serves as an open-access testbed for mobile underwater acoustic networks, combining indoor and lake environments to support both controlled laboratory experiments and realistic field deployments. By incorporating the low-cost, replicable MUR, smaller research groups and educational institutions can now undertake advanced studies in underwater signal processing, networking, and autonomous control. This integration democratizes access to underwater robotics, fosters interdisciplinary collaboration, and enhances experimental reproducibility.

The MUR’s affordability, adaptability, and open-source architecture make it a vital asset for contemporary aquatic research. Its integration into collaborative testbeds like μNet broadens access to underwater robotics and fosters interdisciplinary innovation in marine science, environmental monitoring, and underwater networking. Moreover, the MUR’s flexible design supports a wide array of sensors and communication protocols, allowing researchers to tailor its capabilities to specific experimental needs. By facilitating both laboratory and field experiments, the MUR contributes to the development of scalable underwater systems. In summary, its open-source design, modular framework, and versatile integration options position the MUR as an essential tool for conducting adaptable and scalable underwater research.

## 2 Hardware description

**Fig. 1:**
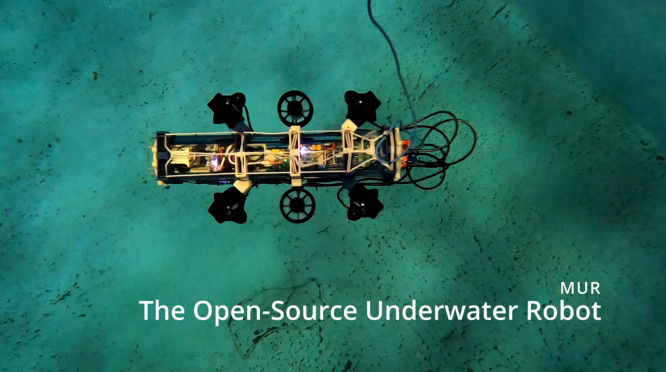
MUR Intro Video – Click to Watch.

**Fig. 2:**
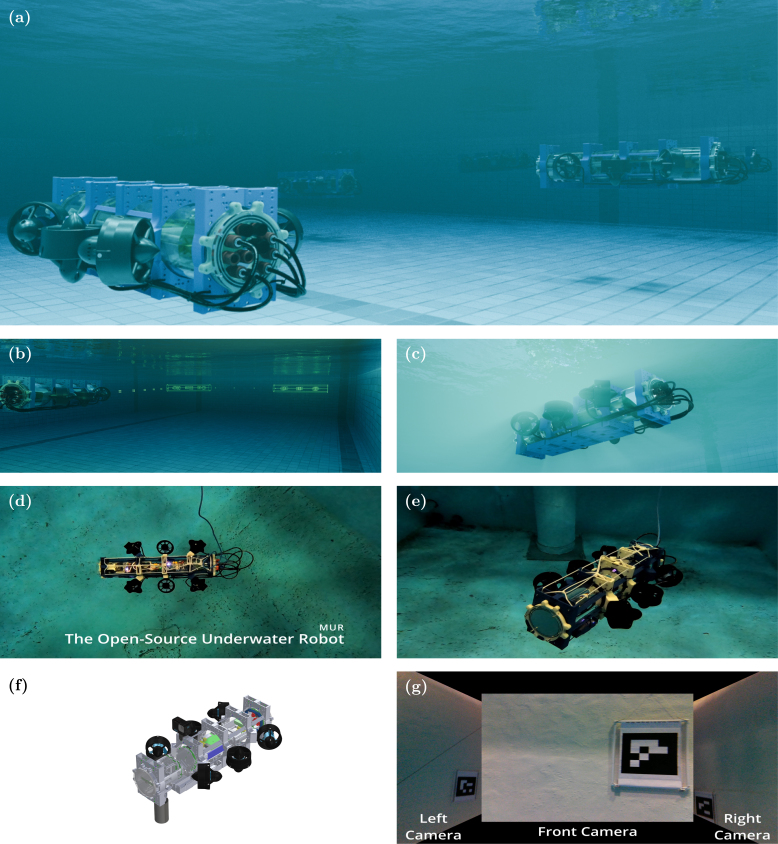
The Miniature Underwater Robot (MUR): (a) Render of MUR swarm, (b) Path planning render, (c) Underbelly render, (d) Underwater photo during operation, (e) Second underwater perspective, (f) CAD model, (g) MUR Camera view with tagged components.

The **Miniature Underwater Robot (MUR)** — Fig. 1, Fig. 2 — is a compact robotic platform that balances cost-effectiveness with advanced capabilities for underwater research. In contrast to conventional Autonomous Underwater Vehicles (AUVs) that rely on proprietary systems, the MUR adopts an open-source hardware and software framework. This design benefits research institutions, hobbyists, and educational programs by reducing costs and providing a modular foundation for underwater exploration.

### 2.1 Design philosophy and architecture

The MUR employs a ROS-based (Robot Operating System) software stack to enable real-time data sharing and streamline the integration of sensors and control algorithms. Modular mechanical and electronic components allow easy reconfiguration, such as swapping thrusters, integrating water-quality sensors, or mounting specialized cameras. This design mitigates the learning curve associated with proprietary or closed-source systems. Further, sensor connectors meet the Pixhawk standards, allowing for a suite of existing sensors to easily be integrated.

Key design elements include a modular 5-degree-of-freedom propulsion system that enables precise position, yaw, and roll control, with passive pitch stability through neutral buoyancy supporting steady station-keeping and dynamic trajectory tracking. The system supports tasks such as station-keeping, trajectory tracking, and a wide range of data collection capabilities. Furthermore, the robot integrates multiple cameras to support perception tasks including visual odometry, object tracking, and machine-learning-based scene interpretation.

The MUR is cost-effective, with prices ranging from $850 to $2000 (cost comparison in Table 4), thereby broadening access for budget-constrained laboratories. Applications include near-shore marine ecology surveys, educational demonstrations, and scientific instrumentation testing.

Conventional AUVs typically employ proprietary hardware and software, which restricts modifications and upgrades. In contrast, the MUR’s open-source, modular architecture facilitates experimentation and collaborative development. Researchers can prototype new control strategies, integrate emerging sensor technologies, and extend the platform’s capabilities for specialized tasks without redevelopment. Thus, the MUR reduces both financial and technical overhead while supporting system adaptation to evolving research needs.

The MUR is scalable to multi-robot systems, enabling cooperative exploration and task allocation among underwater robots. This scalability benefits large-scale environmental monitoring, as multiple units can gather data over extensive areas concurrently. A robust ROS community, combined with comprehensive documentation and open design files, supports the exchange of software modules, troubleshooting methods, and hardware improvements.

### 2.2 Highlights

• **Cost-Effective Research Platform:** Priced between $850 and $2000 (Table 4), it is accessible for smaller institutions and educational programs.
• **Scalable Multi-Robot Deployments:** Supports the operation of multiple units for environmental monitoring, mapping, or swarm experiments.
• **Customizable Open-Source Design:** Facilitates hardware modifications and software configuration to meet specific experimental requirements.
• **Advanced Sensing and Control:** Integrates multiple cameras and supports machine learning pipelines for perception tasks; can operate autonomously or in tethered mode.
• **Educational Resource:** Provides a practical platform for instruction in underwater navigation, machine vision, and open-source development.

Collectively, these features establish the MUR as a robust solution for a range of underwater applications. The combination of affordability, modularity, and strong software support encourages independent research and community-driven advancements. As sensor technologies and control algorithms advance, the open-source architecture of the MUR ensures it remains a valuable resource for underwater exploration and experimentation.

## 3 Design files summary

The documentation of the MUR is given in Table 1. All hardware files are licensed as CERN-OHL-P v2 and all software files are licensed as MIT License. The combined license file is at https://doi.org/10.5281/zenodo.14968240, Path: LICENSE.md.

**Table 1:** Summary of MUR design files.

Design file name	File type	Open-source license	Location ( Zenodo DOI)
MUR_Hardware_Design_File.xlsx	Excel spreadsheet	MIT License	hardware/MUR_Hardware_Design_File.xlsx
CAD models	STEP, SLDPRT, etc.	MIT License	hardware/CAD/
PCB designs and schematics	PCB and Schematic files	CERN-OHL-P v2	hardware/PCBs/
Firmware	Embedded source code (C)	MIT License	hardware/firmware/
MUR_Software_Design_File.xlsx	Excel spreadsheet	MIT License	software/MUR_Software_Design_File.xlsx
Software source code	Source code (Python/C++)	MIT License	software/

### 3.1 CAD

**Fig. 3:**
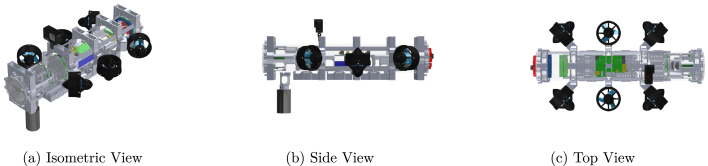
Different views of the Miniature Underwater Robot (MUR) CAD model.

The CAD models (Fig. 3) are provided in SolidWorks format, while 3D-printable files are also available in STL format. Both SolidWorks (*.SLPRT) and STL (*.STL) files are organized within the same folder to facilitate easy identification of parts for printing. See Table 1 for documentation.

### 3.2 Electronic files

Electronic files were designed using EasyEDA and later manufactured and assembled using JLCPCB. The provided files can be uploaded to the EasyEDA platform, granting full access to wiring diagrams, PCB layouts, and Bills of Materials (BOMs). Additionally, all necessary manufacturing files, including the BOM, Pick & Place, and Gerber files for each PCB, are provided separately, allowing for manufacturing without the need to open the project ZIP. See doi.org/10.5281/zenodo.14968240, Path: hardware/PCBs/*/easyeda/Production Files/ for the production files for each PCB. Further, see Table 1 for more documentation.

The Electronic Files consists of (5.1.1) Compute Module Mini, (5.1.2) Sensor and ESC Control Board, and (5.1.3) ESC to Thruster Board. These 3 boards form the main electronic system of the MUR.

### 3.3 Software design files

The software comprises multiple custom ROS packages, each thoroughly documented through in-code comments and README.md files. Documentation is provided both for the complete software suite and for each individual ROS package to ensure clarity and ease of use. For better understanding, each directory in the software suite includes a corresponding README.md file that explains the directory structure, individual files, and overall software organization. See Table 1 for documentation.

Of note, the most effective way to understand the software structure is by exploring the file tree doi.org/10.5281/zenodo.14968240, Path: software/* (along with the paired README.md files).

#### 3.3.1 ROS packages summary

The MUR software suite primarily consists of 4 ROS packages: (1) mur, (2) mur_control, (3) mur_model, and (4) mur_sensors.

The **mur ROS Package** acts as the central hub for the Miniature Underwater Robot (MUR) system. It coordinates the initialization of all sub-components by providing a unified launch file (mur.launch) that loads essential configuration files such as global_config.yaml and environment_info.yaml. These files establish communication parameters, module information, and environmental settings that are critical for proper system operation. By streamlining the launch process and enabling easy customization (for instance, the inclusion of optional modules by simply uncommenting a line), this package ensures that all sensor, model, and control nodes are started seamlessly, laying a solid foundation for the entire MUR ecosystem.

The **mur_control ROS Package** is dedicated to managing the robot’s actuation and stabilization mechanisms. It offers a set of scripts that blend manual keyboard inputs with PID-based automated control to generate and refine pose setpoints. The control package is configured via a YAML file (control_info.yaml) that allows users to adjust key parameters such as PID gains, control loop frequencies, and manual thruster update rates. This modular control architecture not only facilitates precise thruster management and dynamic stabilization but also incorporates emergency stop and reset functionalities to guarantee safe operation during both manual and automated maneuvers.

The **mur_model ROS Package** provides a framework to model thrusters, mechanics, and other physical properties. It also handles command-to-thruster inputs, camera management, localization conversion, and TF2 broadcasting. The configuration files (such as model_info.yaml and aruco_info.yaml) detail sensor calibrations, robot structure, and thruster mechanics.

The **mur_sensors Package** is responsible for the acquisition, processing, and integration of diverse sensor data, which is critical for accurate underwater navigation and environmental monitoring. It supports a variety of sensors, including cameras, IMUs, pressure sensors, and magnetometers, and employs sensor fusion algorithms to enhance the reliability of the data. Camera connections are launched here, with their physical parameters recorded in the **mur_model ROS Package**. Additionally, the package implements dynamic network configuration to automatically manage IP addresses and communication ports, ensuring seamless connectivity between the sensor modules and the ROS framework. By setting up dedicated ROS publishers and subscribers, it facilitates real-time data processing and visualization, thereby ensuring that accurate and timely sensor information is available to the other components of the MUR system.

## 4 Bill of materials summary

The full BOM documentation is available in Table 2. A succinct summary is provided in Table 3 for easy reading. Of note, there are 4 possible build options, as made clear in Table 4.

**Table 2:** Bill of Materials (BOM) documentation.

Designator	Component	Qty	Cost/unit (USD)	Total (USD)	Source of materials	Material type
See BOM: hardware/BOM/MUR_BOM.xlsx. Summary in Table 3.

**Table 3:** Summary of Bill of Materials (BOM) with cost breakdown.

Component	Cost (USD)	Units	Total
**Frame options (Choose one option):**			
Hardware w/o thrusters (Self-3D printing)^a^	$428.15	1	$428.15
Hardware w/o thrusters (Commercial printing)^a^	$589.12	1	$589.12

**Thruster options (Choose one option):**			
Off-the-shelf thrusters (Thrust Option 1)^b^	$30.00	6	$180.00
T200 thrusters (Thrust Option 2)^b^	$200.00	6	$1200.00

**Electronics:**			
Compute PCB - Components, Assembly, & Manufacture^c^	$58.13	1	$58.13
Sensor PCB - Components, Assembly, & Manufacture^c^	$35.29	1	$35.29
ESC breakout PCB - Components, Assembly, & Manufacture^c^	$17.12	1	$17.12
Teensy 4.0	$30.00	2	$60.00
Raspberry Pi CM4	$80.00	1	$80.00

a Print Option 1 and Print Option 2 are mutually exclusive; only one should be selected.

b Thrust Option 1 and Thrust Option 2 are mutually exclusive; only one should be selected.

c Items with separate BOMs for subcomponents. Manufacturing and assembly was done externally, so costs may vary. This table represents a summary of the full BOM.

**Table 4:** Cost comparison for the different build options.

	Off-the-shelf thrusters	T200 thrusters
Self 3D printing	$858.69	$1878.69
Commercial 3D printing	$1019.66	$2039.66

## 5 Build instructions

The build consists of: (5.1) manufacturing the requisite PCBs; (5.2) flashing firmware; and (5.3) hardware assembly.

### 5.1 PCB manufacturing and assembly

The PCB manufacturing and assembly process consists of three main components: the (5.1.1) Compute Module Mini; the (5.1.2) Sensor and ESC Control Board; and the (5.1.3) ESC to Thruster Board. These boards were manufactured and assembled in a factory and are not intended for manual assembly. Files and full documentation provided in Table 1.

#### 5.1.1 Compute Module Mini

##### 5.1.1.1 Overview

**Fig. 4:**
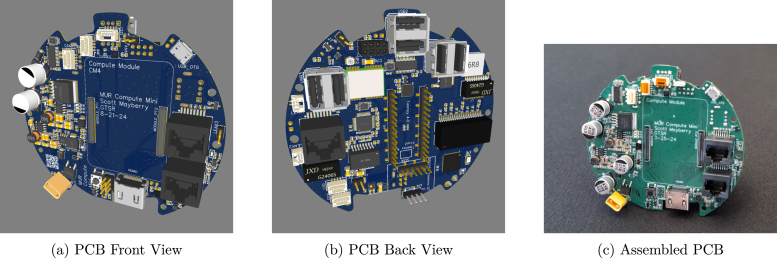
Views of the Compute module mini PCB, including front, back, and a real-life assembled version.

The MUR Compute Module Mini PCB (Fig. 4) integrates a wide array of sensors, interfaces, and power management features to enable robust environmental interaction and sensing. The sensor suite on this board includes the following devices:

1. **Magnetometers:** HSCDTD008A (ALPSALPINE, 3-axis, compass orientation) and LIS2MDLTR (STMicroelectronics, 3-axis, precise measurement).
2. **Accelerometer:** KXTJ3-1057 (ROHM Semiconductor, 3-axis for acceleration).
3. **IMUs:** LSM6DS3TR-C (STMicroelectronics, 6-DOF combining gyroscope and accelerometer), MPU6500 (InvenSense, 6-axis integration), and BNO055 (STEMMA QT Board) (Bosch Sensortec/Adafruit, 9-DOF absolute orientation).
4. **Environment Sensors:** AHT20 (Guangzhou Aosong Electronics, temperature and humidity) and DPS310 (Infineon, barometric pressure).
5. **GPS:** ATGM332D-5N31 (ZHONGKEWEI, geolocation).

Connectivity is achieved through a variety of interfaces. Dedicated I2C ports provide one connection for the RPi Compute Module 4 (CM4) and two additional ports for extra sensors or peripherals, while a USB OTG port facilitates flashing of the CM4. The board also includes two CAN bus interfaces for communication with external control units, six USB 2.0 ports for peripheral connectivity, and three leak detection inputs via JST connectors and a pin header, in addition to a dedicated 5 V fan port for thermal management.

Communication capabilities are provided by the CM4’s built-in WiFi, an integrated three-port Ethernet switch for stable wired connectivity, and a dedicated CC1101 radio module slot supporting sub-1 GHz transmissions for remote control and data transfer. Power input is handled via XT60 connectors for high-current applications and XT30 outputs for lower current subsystems, all powered by a 4S Lipo battery. For more details, see doi.org/10.5281/zenodo.14968240, Path: hardware/PCBs/mur_compute_module_mini_pcb/README.md.

##### 5.1.1.2 Build instructions

The PCB should be manufactured commercially (not hand built). For assembly into the MUR, please see Section 5.6.

#### 5.1.2 Sensor and ESC control board

##### 5.1.2.1 Overview

**Fig. 5:**
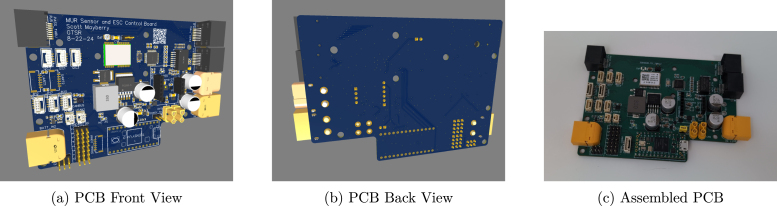
Views of the sensor and ESC Control board, including front, back, and a real-life assembled version.

The MUR Sensor and ESC Control PCB provides more sensor data and dedicated ESC control, following the PixHawk I/O standard for integration with robotics and UAV systems. It also incorporates the same sensor suite as the Compute Module Mini (see Fig. 5).

The duplication of the sensor package serves several purposes: multiple IMUs provide redundant references for determining vehicle orientation; distributing IMUs across the vehicle mitigates errors caused by magnetic interference when sensors are located near high-power components, allowing users to dynamically select IMUs that are furthest away from these high-power sources; accelerometers and gyroscopes at different positions yield more precise measurements of positional changes, improving localization accuracy; the total cost of adding full redundancy is less than $10 USD, making it a cost-effective way to enable backup sensing and sensor fusion across the vehicle; placing inexpensive sensor suites in multiple locations gives users the flexibility to expand or relocate sensors depending on design needs; and finally, the approach simplifies testing, since sensor data can be collected from either the Compute Module Mini or the Sensor and ESC Control Board without requiring the entire system to be active.

Connectivity is achieved through 3 I2C ports, 2 serial ports, and 3 CAN bus interfaces, along with leak detection via 2 JST connectors and 3 header pins, and 9 PWM outputs (8 for servo control and 1 for batch control) for actuator management. Wired communication utilizes a W5500 chip with an RJ45 connector, and power is supplied by a 4S Lipo battery via XT60 (high-current) and XT30 (low-current) connectors. This integrated design ensures proper sensor acquisition, ESC control, and reliable interfacing with external systems. For more details, please see doi.org/10.5281/zenodo.14968240, Path: hardware/PCBs/mur_sensor_and_esc_control_pcb/README.md.

##### 5.1.2.2 Build instructions

The PCB should be manufactured commercially (not hand built). Files provided in Table 1. For assembly into the MUR, please see Section 5.5.

#### 5.1.3 ESC to Thruster Board

##### 5.1.3.1 Overview

**Fig. 6:**
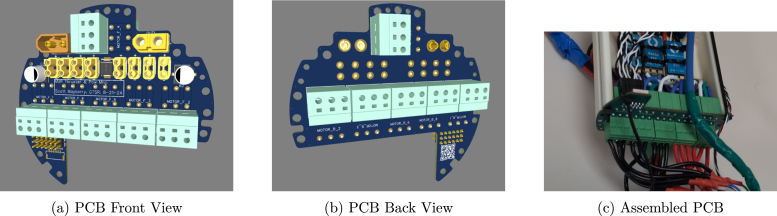
Views of the ESC to Thruster PCB, including front, back, and a real-life assembled version.

This board manages ESC power and connects the external thrusters to the Sensor and ESC Control board. It provides power output through XT60 connectors for high-current needs, while 8 dedicated XT30 connectors supply power to the ESCs. Additionally, spring terminals are implemented to allow thrusters to be plugged in without traditional connectors, enabling easy removal or swapping of penetrators when maintenance or replacement is required. For more details, please see doi.org/10.5281/zenodo.14968240, Path: hardware/PCBs/mur_esc_to_thruster_pcb/README.md (see Fig. 6).

##### 5.1.3.2 Build instructions

The PCB should be manufactured commercially (not hand built). Files provided in Table 1. For assembly into the MUR, please see Section 5.7.

### 5.2 Firmware installation

The firmware is divided into two sections: Compute Module Mini Setup (Section 5.2.1) and Teensy Setup (Section 5.2.2). The compute module mini requires flashing the board, and installing some custom services for camera streaming and IP broadcasting. The Teensy setup involves compiling the given *.ino file and modifying the config.h file in the Teensy firmware directory.

#### 5.2.1 Compute Module Mini Setup

This section covers the Compute Module Mini, and the three steps required for functionality (Table 5):

##### 5.2.1.1 Flashing the CM4

1. **Step 1: Flash the CM4 module.** If the Compute Module 4 (CM4) is not already flashed, follow the recommended tutorial: https://www.youtube.com/watch?v=jp_mF1RknU4. This will prepare your module for further configuration.

##### 5.2.1.2 Camera streaming setup

1. **Step 1: Update and Install Dependencies**

**Figure:**
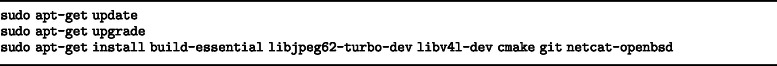
2. **Step 2: Download and Compile mjpg-streamer**

**Figure:**
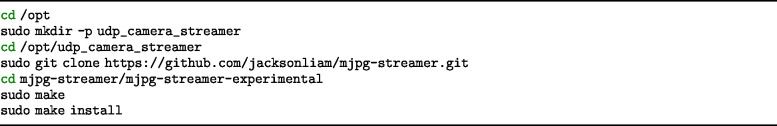
3. **Step 3: Confirm Installation** Run:

**Figure:**
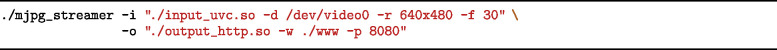

Then visit http://<Raspberry_Pi_IP>:8080 to view the stream.
4. **Step 4: Install USB ID Path Tag Identifier** Create the bash script:

**Figure:**
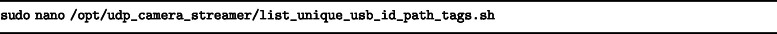

and copy the code from doi.org/10.5281/zenodo.14968240, Path: hardware/firmware/compute_module/ service_setup/camera_streaming/list_unique_usb_id_path_tags.sh
5. **Step 5: Set Up the Camera Streaming Service** Create the Python file:

**Figure:**
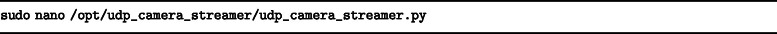

and copy in the Python code from doi.org/10.5281/zenodo.14968240, Path: hardware/firmware/compute_module/ service_setup/camera_streaming/udp_camera_streamer.py Then, create the systemd service file:

**Figure:**
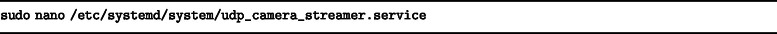

Insert the following configuration:

**Figure:**
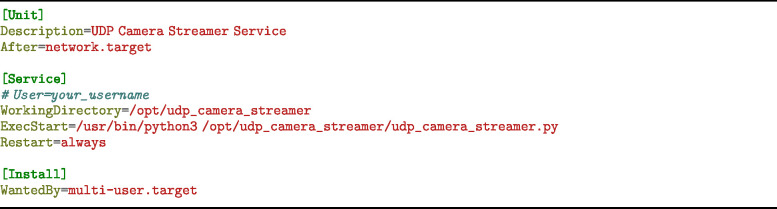

Reload and enable the service:

**Figure:**
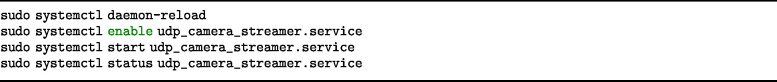

##### 5.2.1.3 Broadcast IP setup

1. **Step 1: Install Necessary Packages**

**Figure:**
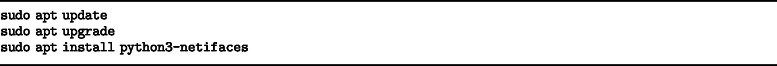
2. **Step 2: Set Up the Broadcast Service** Create the directory and Python file:

**Figure:**
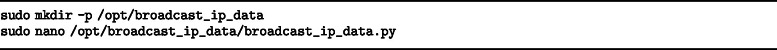

Then copy the Python code from doi.org/10.5281/zenodo.14968240, Path: hardware/firmware/compute_module/ service_setup/broadcast_ip/broadcast_ip_data.py Next, create the systemd service file:

**Figure:**
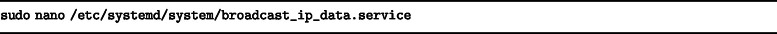

Insert the following configuration:

**Figure:**
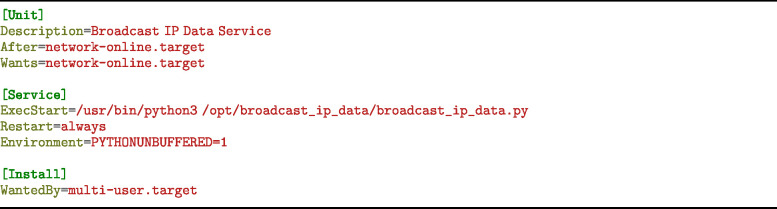
3. **Step 3: Reload systemd and Enable the Service**

**Figure:**
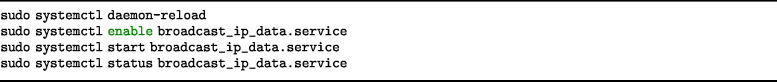

**Table 5:** Compute Module 4 setup guides.

Guide	Description	Source	Zenodo, doi.org/10.5281/zenodo.14968240
Flashing the CM4	Flash the Compute Module if it is not already flashed.	External YouTube Tutorial	N/A

Camera streaming	Auto-launch USB cameras and stream video over Ethernet.^a^	Camera Streaming Guide	Path: hardware/firmware/compute_module/service_setup/ camera_streaming/camera_stream_setup_guide.md

Broadcast IP	Broadcast the device’s IP address for auto ROS node discovery.	Broadcast IP Guide	Path: hardware/firmware/compute_module/service_setup/ broadcast_ip/broadcast_ip_setup_guide.md

a The camera streaming service, outlined below, works for any USB camera type. The MUR uses Arducam 8MP IMX219, and the service works by matching each camera’s unique ID_PATH_TAG identifier, ensuring consistent device recognition across reboots and allowing the correct video device to be launched with the specified resolution, frame rate, and stream port via a simple JSON command sent over UDP. This setup can be easily modified to instead match other parameters loaded from the camera if this unique id is not sufficient to uniquely identify different cameras and pair them to a stream.

#### 5.2.2 Teensy setup

The Teensy Setup section details the steps required to flash and configure the firmware for the Teensy microcontroller used in the Miniature Underwater Robot (MUR). There are corresponding Teensy board slots on the Compute Module Mini board and the Sensor and ESC Control Board. This firmware handles critical tasks such as sensor interfacing, actuator control, and communication protocols, complementing the core MUR firmware.

Two Teensy boards needs to be flashed. Using the Arduino IDE, with the Teensyduino add-on, using a USB cable, flash the *.ino file to the boards.

##### 5.2.2.1 Teensy setup

1. **Step 1: Install Arduino and Teensyduino** Install these on your local computer.
2. **Step 2: Set Up the Config File** In the config.h file, please set the board type (fuselage or compute module mini paired) by uncommenting proper line.

**Figure:**
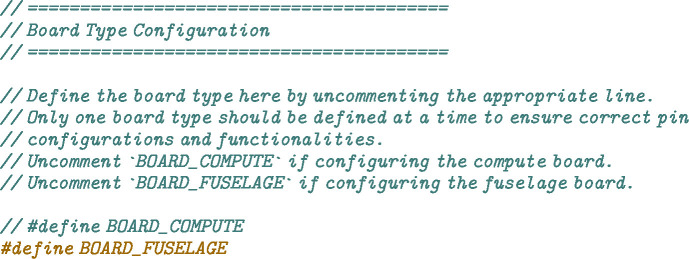
3. Step 3: Flash the *.ino file to the board. (repeat for other board)

### 5.3 Hardware assembly

The assembly involves constructing multiple subcomponents, with build videos and instructions provided in the following subsections. The full BOM is listed in Table 2. For PCBs, this guide assumes they have been factory-assembled, requiring no additional assembly (Section 5.1). This guide covers the following areas:

• **Thruster Assemblies** (Section 5.4)
• **Sensor and Battery Cradle Assembly** (Section 5.5)
• **Camera & Compute Cradle Assembly** (Section 5.6)
• **ESC Cradle Assembly** (Section 5.7)
• **Ballast Box Assembly** (Section 5.8)
• **Final Full Assembly** (Section 5.9)

Please follow each section carefully to ensure proper assembly and system functionality. Of note, we have minimized the number of bolts required to assemble the MUR by instead relying on zip ties for easy mounting. This has functioned well in practice.

All video files are also provided in the repository doi.org/10.5281/zenodo.14968240, Path: hardware/Assembly/ assembly_videos/**. The assembly is also available in the README.md at Path: hardware/Assembly/README.md.

### 5.4 Thruster assemblies

**Fig. 7:**
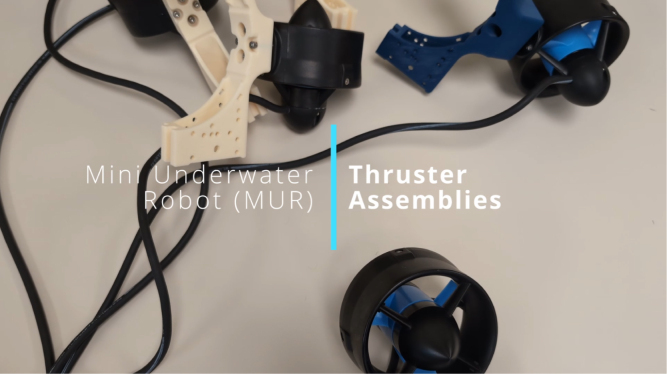
Thruster Assembly Video - Click to Watch.

The thruster assembly process involves three steps: printing the thruster mounts, securely attaching the thrusters to the mounts, and applying a rubber seal to achieve a friction press fit. These procedures ensure both mechanical integrity and effective vibration damping. Please see Fig. 7 for visual assembly.

#### 5.4.1 Fabrication of components

Begin by printing the required thruster mounts from the Zenodo repository (hardware/CAD/Thrusters and Assemblies/):

1. thruster_mount_blank.STL (Qty: 4) – Blank mount with no thruster mounting.
2. thruster_mount_0_degree.STL (Qty: 2) – Mount with a 0-degree orientation.
3. thruster_mount_45_degree.STL (Qty: 4) – Mount with a 45-degree orientation.

#### 5.4.2 Attach thrusters to mounts

Once the mounts are ready, secure each thruster to its designated mount using the stainless steel bolts. Tighten these bolts uniformly to maintain proper alignment while avoiding excessive torque that could strip the threads or cause structural stress to the 3D printed mounts.

#### 5.4.3 Apply rubber seal and finalize assembly

Before mounting the thruster assemblies onto the vehicle frame, apply the rubber seals between the thruster mounts and the frame. These seals provide a friction press fit that enhances grip and minimizes tolerancing issues.

### 5.5 Sensor and Battery Cradle Assembly

**Fig. 8:**
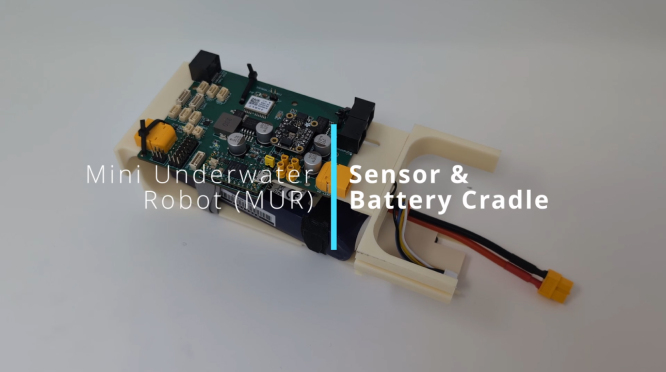
Sensor and Battery Cradle Assembly Video - Click to Watch.

The Sensor and Battery Cradle subassembly integrates sensor data and power management into the MUR system. Its fabrication involves producing several components from the provided CAD files, followed by a simple assembly to secure all connections. Please see Fig. 8 for visual assembly.

#### 5.5.1 Fabrication of components

Begin by printing the required Sensor Battery and ESC Cradle components from the Zenodo repository (hardware/CAD/ Cradles/Sensor Battery and ESC Cradle/battery_and_bluebuzz_cradle):

1. battery_and_sensor_pcb_holder.STL (Qty: 1)
2. battery_and_sensor_pcb_holder_ring_mount.STL (Qty: 1)

Additional references for assembly include the CAD Reference Assembly (sensor_battery_esc_cradle.SLDASM in hardware/CAD/Cradles/Sensor Battery and ESC Cradle), which serves as a visual guide for assembly and part verification.

#### 5.5.2 Assemble the Cradle

Once all parts are printed, proceed with the assembly:

1. **Attach PCB to Top Bracket:** Secure the PCB to the top bracket using zip ties, ensuring a firm yet non-compressive connection.
2. **Mount Battery:** Fasten the battery to the base using reusable Velcro straps for stability and easy future replacement.
3. **Cable Management:** Route and connect the required cables (Ethernet, USB, power, and motor control) neatly to minimize strain and prevent interference.

### 5.6 Camera & Compute Cradle Assembly

**Fig. 9:**
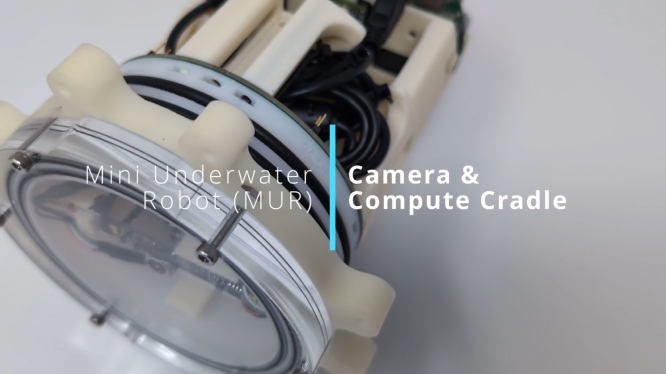
Camera and Compute Cradle Assembly Video – Click to Watch.

This section details the assembly of the Camera & Compute Cradle, a subassembly that integrates the imaging and computational modules for the MUR. The following steps outline the proper sequence to ensure a secure, watertight installation of the camera system and its associated components. Please see Fig. 9 for visual assembly.

#### 5.6.1 Fabrication of components

Begin by printing the required components using the provided files in the Zenodo repository:

• **From hardware/CAD/Cradles/Compute and Camera Cradle:**
  – camera_and_compute_print.STL (Qty: 1)
  – camera_to_compute_bridge_print.STL (Qty: 1)
• **From hardware/CAD/Cradles/Compute and Camera Cradle/compute_mini:**
  – mur_compute_mini_case_back.STL (Qty: 1)
  – mur_compute_mini_case_front.STL (Qty: 1)
• **Sealed Flange (from hardware/CAD/Cradles/Cradle End Cap Assembly):**
  – sealed-flange-trip-seal-tighter-inner.STL (Qty: 1)
• **For the Face Plate (from hardware/CAD/End Caps/Flat):**
  – end_cap_blank.SLDPRT (Qty: 1) - this part should be laser cut or purchased.

#### 5.6.2 Assemble the Cradle

Once all parts are fabricated:

• Secure the PCB to mini case back and front utilizing zip ties.
• Further secure the PCB to the camera camera_to_compute_bridge_print.STL. This will bridge between the Compute Module Mini and the Cameras.
• Attach the camera_to_compute_bridge_print.STL to the camera_and_compute_print.STL using the alignment holes and zip ties.
• Install the cameras by inserting them into their designated slots on the camera_and_compute_print.STL. These will be the radial bars that form both the structure as well as have mounting for the cameras. Fasten them with zip ties so they remain securely in place without obstructing their fields of view.

#### 5.6.3 Finalize the Cradle Assembly

• Route and secure all cables neatly to avoid interference with camera views.
• Install the O-rings (see details in hardware/CAD/Cradles/Cradle End Cap Assembly/o_rings/) onto the sealed-flange-trip-seal-tighter-inner.STL.
• Mount the face plate, ensuring proper alignment with the rest of the assembly and a tight seal.

For further mounting guidance and visual assembly verification, refer to the CAD reference files b0441_usb_camera.SLDPRT and compute_and_camera_cradle_assembly.SLDASM (both located in hardware/CAD/Cradles/Compute and Camera Cradle). Following these steps, and using these CAD models as a guide, will yield a Camera & Compute Cradle Assembly ready for integration into the MUR system.

### 5.7 ESC Cradle Assembly

**Fig. 10:**
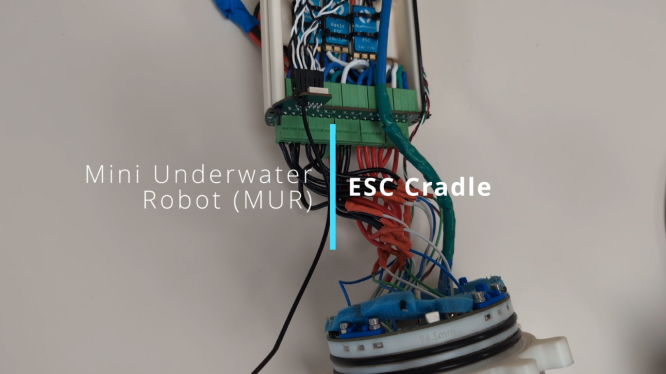
ESC Cradle Assembly Video – Click to Watch.

This section describes the step-by-step process for assembling the ESC Cradle, which is designed to securely mount the PCB and ESCs and establish connections for the ESCs, thrusters, and peripheral penetrators (for thruster wiring, sensors, and the Ethernet tether). Follow the instructions carefully to ensure reliable operation and ease of maintenance. Please see Fig. 10 for visual assembly.

#### 5.7.1 Fabrication of components

Begin by fabricating the required components from the provided files, located in the Zenodo repository (Path: hardware/CAD/Cradles/Sensor Battery and ESC Cradle/esc_assembly_cradle):

1. **3D Printed Components (from .../esc_assembly_cradle):**
  (a) esc_vert_printed_casing.STL (Qty: 1)
  (b) esc_vert_printed_casing_backpiece.STL (Qty: 1)
2. **Sealed Flange (from hardware/CAD/Cradles/Cradle End Cap Assembly):**
  (a) sealed-flange-trip-seal-tighter-inner.STL (Qty: 1)
3. **Laser-Cut End Cap (from hardware/CAD/End Caps/Flat):**
  (a) end_cap_M10x10,M14x1.DXF (Qty: 1)

#### 5.7.2 Assemble the Cradle

With the printed parts ready, secure the PCB to the front of the 3D-printed casing using zip ties, ensuring a firm yet non-compressive attachment. Mount the ESCs to the body of the cradle, and wire the connections to the corresponding terminals (marked on the PCB).

#### 5.7.3 End cap penetrator assembly

This assembly assumes that you have potted the thrusters and sensors using an epoxy mix with the listed penetrators. If not, please see any penetrator potting guide for assistance. Assemble the end cap by installing the penetrators required for routing thruster wiring, sensor cables, and the Ethernet tether through the designated openings in the end cap:

• Align the penetrators with their corresponding openings in the end cap.
• Secure the penetrators using the included screw-on plate to guarantee a stable, leak-proof installation.
• Apply the O-ring to the sealed flange to provide a watertight seal before mounting the end cap onto the ESC cradle.
• Make sure to properly pot the penetrators for water tight sealing.

#### 5.7.4 Finalize the Cradle Assembly

Carefully connect the thrusters by inserting its connectors into the designated terminals, ensuring every connection is secure. These can be removed by depressing the lever in the terminal for easy thruster replacement.

For further mounting guidance and visual assembly verification, refer to the CAD assembly reference file esc_assembly_ cradle.SLDASM.

### 5.8 Ballast Box Assembly

**Fig. 11:**
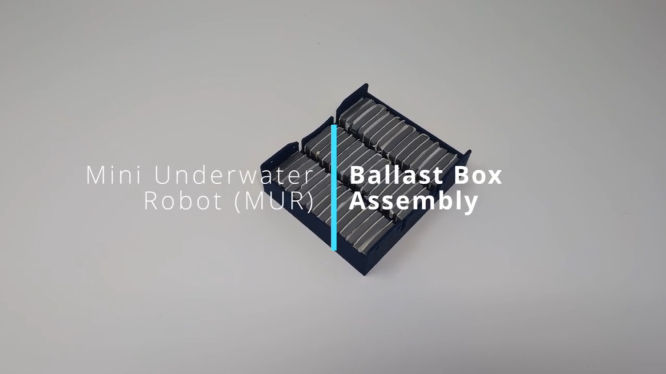
Ballast Box Assembly Video – Click to Watch.

This section details the assembly process for the Ballast Box, which is critical for achieving the desired buoyancy and balance of the MUR. Follow the instructions carefully to ensure correct installation and secure attachment of the ballast weights. Please see Fig. 11 for visual assembly.

#### 5.8.1 Fabrication of components

Begin by printing the Ballast Box from the Zenodo repository (hardware/CAD/Ballast Box/ballast_box.STL) (Qty: 3).

#### 5.8.2 Assemble the Ballast Box

Evenly distribute the 1oz wheel weights within each box to achieve neutral buoyancy. It may require a few trials to ballast the MUR properly. Secure the weights using zip ties to prevent any movement during operation. Verify that each weight is firmly attached to maintain consistent ballast.

The current ballast box is secured to the base using zip ties, which serves as a temporary but suboptimal solution. A key priority for future development of the MUR is the design of an improved ballast mechanism — such as a swappable snap-on module or slide-on mounting system — that enables rapid adjustment and re-ballasting across different environments. While the present approach is adequate for repeated deployments in the same setting once initial ballast adjustments are made, it lacks the flexibility and robustness required for broader use. Enhancing this system will significantly streamline field preparation and improve the overall usability of the platform.

### 5.9 Final Full Assembly

**Fig. 12:**
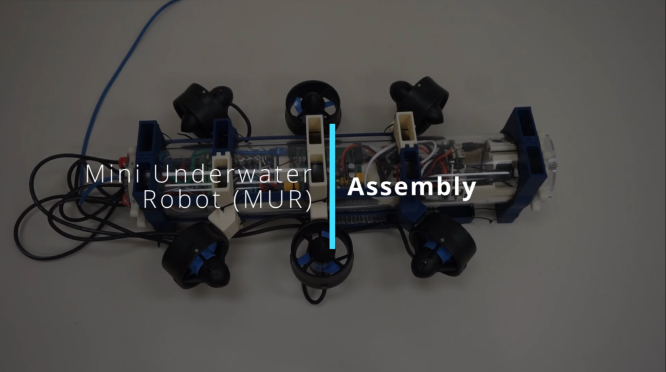
Final Full Assembly Video – Click to Watch.

This section outlines the final assembly process for the complete MUR system, integrating all subassemblies to ensure that the robot is fully operational and ready for deployment. Follow these steps carefully, and refer to Fig. 12 for visual guidance.

#### 5.9.1 Gather and prepare components

Collect all necessary components:

• Main acrylic tube, ballast boxes, and thruster brackets.
• Spacers (see fabrication below), along with Ethernet, USB, and power cables.
• ESC cradle, compute cradle, and battery/sensor cradle subassemblies.
• Zip ties and other fasteners.

#### 5.9.2 Fabrication of additional components

If not already fabricated, print the additional parts required for the final assembly:

1. **From hardware/CAD/Thrusters and Assemblies:**
  (a) thruster_spacer.STL (Qty: 20)

#### 5.9.3 Assemble the full system

• **Position Thruster Brackets:** Attach the thruster brackets to the main structural tube. Secure them with zip ties loosely to allow for later adjustments.
• **Insert Spacers:** Install the printed spacers into their designated locations on the thruster mounts and secure them with additional zip ties to ensure proper alignment.
• **Install Ballast Boxes:** Place the ballast boxes in their assigned positions to maintain neutral buoyancy.
• **Mount the Camera & Compute Cradle Assembly:** Insert the Camera & Compute Cradle Assembly, ensuring that the Ethernet, USB, and power cables are routed correctly.
• **Insert Sensor and Battery Cradle Assembly:** Insert the Sensor and Battery Cradle Assembly, verifying that all electrical connections are properly made.
• **Mount the ESC Cradle Assembly:** Mount the ESC Cradle Assembly, ensuring that all wiring connections (including those for thrusters) are correctly assigned.
• **Seal the Assembly:** Once all subassemblies are in place, seal the main tube.

#### 5.9.4 Finalize and verify assembly

• Organize and secure all cables using zip ties to prevent interference.
• Perform a thorough system check to verify that all mechanical and electrical connections are secure.
• Before deployment, we recommend a ballast check (may require re-ballasting), and a vacuum test.
• Confirm that the overall assembly is complete and that the MUR is ready for deployment.

For further mounting guidance and visual assembly verification, refer to the full CAD assembly hardware/CAD/mur_ assembly.SLDASM.

## 6 Operation instructions

This section provides a detailed guide for safely launching and operating the Miniature Underwater Robot (MUR). This section assumes you have (1) installed ROS, (2) assembled the MUR, (3) installed the proper firmware, and (4) installed the ROS packages. This is guide is for launching in tethered mode. Launching untethered requires an acoustic modem, specifically the BlueBuzz modem, for communication [14] and is still in development. The MUR utilizes ROS1 currently, with migration work in progress for ROS2.

### 6.1 Launching the system

The entire MUR system is launched with a **single command**, which initializes all ROS packages and loads the necessary configuration files.

1. Power on the MUR by connecting the battery and sealing the tube. Connect the MUR to an Ethernet switch that is on the same network as your control computer.
2. (Optional) Configure sensors and controller parameters. Defaults are already set (Section 6.2).
3. Ensure roscore is running on your control computer. In a terminal, run

**Figure:**
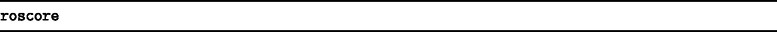
4. Execute the following command (in a different terminal) to start the entire system:

**Figure:**
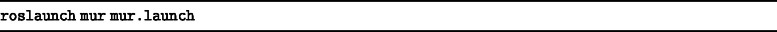

The system will load all configurations and initialize sensor, model, and control nodes. **The system now can be controlled with keyboard, and is recording all camera and sensor data.**

Following these operational guidelines will ensure that the MUR system runs safely and efficiently, enabling reliable underwater exploration and data collection.

### 6.2 (Optional) software configuration

The MUR system is powered by four primary ROS packages that collectively manage system configuration, sensor data processing, control command generation, and physical constraints. Each package is configured using YAML files, ensuring a flexible and customizable setup. Global and environmental settings are specified in global_config.yaml and environment_info.yaml (within the core mur package), control parameters in control_info.yaml (in the mur_control package), and sensor settings in sensor_config.yaml (in the mur_sensors package). README.md with detailed implementation and commented code are located in doi.org/10.5281/zenodo.14968240, Path: software/ROS_packages/src/

#### 6.2.1 MUR sensors package

The mur_sensors package provides real-time environmental perception. It acquires and fuses data from a variety of sensors (e.g., cameras, IMUs, pressure sensors, leak detectors) and uses predefined communication settings (such as comm_port 51 585 and broadcast_port 51584) to reliably transmit sensor data. Sensor modules are pinged every 3 s via port 8888 to maintain active connections, while data is received over UDP on a designated port. The package’s launch file loads these settings and starts nodes for sensor reception, IMU fusion, and network configuration. README.md with detailed implementation and commented code are located in doi.org/10.5281/zenodo.14968240, Path: software/ROS_packages/src/mur_sensors/.

#### 6.2.2 MUR control package

The mur_control package is responsible for generating and managing the robot’s control commands. It processes manual inputs (such as keyboard commands) and blends them with PID-based corrections, using parameters defined in control_info.yaml (including update frequency, PID gains, and control modes). Nodes launched by this package compute thruster commands based on the combined inputs, ensuring responsive and stable operation. README.md with detailed implementation and commented code are located in doi.org/10.5281/zenodo.14968240, Path: software/ROS_packages/src/mur_control/.

#### 6.2.3 MUR model package

The mur_model package contains the robot’s physical dynamics and provides a digital representation of the MUR. Control and operation is based on this model. It defines key physical properties — such as mass, inertia tensor, and coordinate frames — in model_info.yaml, and specifies thruster configurations including positions, orientations, and ESC settings. The package launches nodes for thruster command conversion and dynamic transform broadcasting, facilitating comprehensive simulation and testing. README.md with detailed implementation and commented code are located in doi.org/10.5281/zenodo.14968240, Path: software/ROS_packages/src/mur_model/.

#### 6.2.4 MUR core package

The core mur package integrates system-wide configurations and launches all sub-packages. It loads global parameters (including module information and communication settings) from global_config.yaml and environmental parameters (such as fluid type, density, and gravitational constant) from environment_info.yaml. A unified launch file synchronizes the startup of the sensor, model, and control packages, ensuring coordinated operation across the entire system. README.md with detailed implementation and commented code are located in doi.org/10.5281/zenodo.14968240, Path: software/ROS_packages/src/mur/

## 7 Validation and characterization

**Fig. 13:**
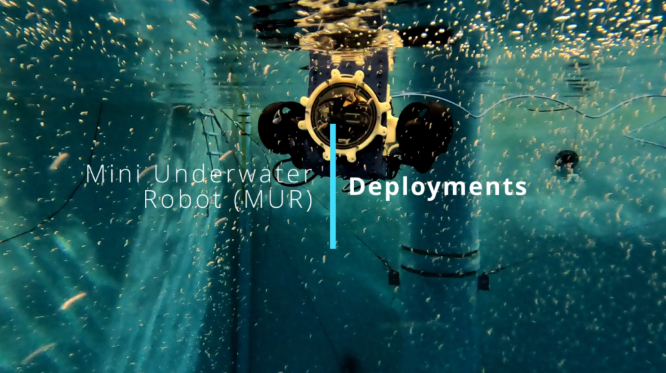
Example deployment video – Click to Watch.

To demonstrate the operation of the MUR – Intro Video in Fig. 1 and Example Deployments in Fig. 13 – and characterize its performance, we conducted a series of experiments in a controlled water tank, focusing on station-keeping accuracy, power consumption, and communication. These trials explored a variety of underwater research conditions, including: manual control, camera-based navigation for image processing tasks, and trajectory tracking. The tests demonstrated the MUR’s capacity for reliable maneuvering, ease of deployment, and ability to collect data in an aquatic environment. In the video, an additional rope is attached to the top of the MUR as a carrying handle, since the waterline in the test tank is low. This rope provides a convenient grip for removing the MUR between deployments. Such modifications are straightforward to implement using the numerous mounting points available on the MUR.

### 7.1 Example use case: Underwater station-keeping in a test tank

One illustrative example involved commanding the MUR to maintain a fixed position at varying depths. The robot’s onboard PID controllers used inputs from integrated inertial and pressure sensors to make real-time adjustments to the thrusters. Fiducial markers were placed in the tank and anchored to the bottom. Video streams from the camera subsystem utilized these fiducial markers for localization, using the Open-CV ArUco tag detection library . Over a 15-minute trial, the MUR maintained its position. Additionally, the robot communicated sensor data back to a base station via a tether, allowing operators to observe and record performance metrics in real time.

#### 7.1.1 Performance metrics

In our characterization, we measured several operational parameters relevant to typical research deployments:

• **Battery Endurance:** Using a standard 4S (3200 mAh) lithium-ion battery pack, the MUR operated for approximately 80 min under moderate thruster use. This duration can vary based on thruster power consumption.
• **Station-Keeping Accuracy:** In a test tank with minimal currents, the MUR maintained position within ±20cm over a 15-minute interval, demonstrating effective PID-based stabilization. This included positioning at multiple different depths between 1–10 m.
• **Depth Tracking:** When updating the target depth during experimentation, the MUR properly generated a trajectory and smoothly transitioned to the new depth.

#### 7.1.2 Capabilities and limitations

• **Multi-Robot Scalability:** The open-source software architecture allows for straightforward networking of multiple MUR units, enabling cooperative tasks such as synchronized sampling or formation-based navigation.
• **Flexible Sensor Integration:** The modular layout and ROS-based system make it simple to mount additional sensors (e.g., water-quality probes, extra cameras) or replace existing modules.
• **Camera-Based Localization:** Multiple camera feeds support computer vision pipelines for object tracking or visual odometry, though highly turbid water may reduce camera-based accuracy.
• **Operational Depth Range:** Recommended for shallow deployments (0–10 m). Deeper operations are possible with additional waterproofing and pressure-rated components.

Overall, these validation trials confirm that the MUR meets the core objectives of affordability, modularity, and performance for shallow water studies. Researchers can further tailor the platform to specific tasks, taking advantage of the open-source ROS framework and hardware extensibility to adapt the MUR to new and evolving experimental needs.

## 8 Future work

Future development of the MUR will focus on improving both functionality and ease of deployment. A central priority is the redesign of the ballast system to move beyond the current provisional solution and provide a robust, field-ready alternative. Concepts under consideration include modular snap-on ballast units and slide-on mounting tracks, both of which would allow rapid reconfiguration and simplified re-ballasting across diverse environments. A more versatile and durable ballast system will substantially reduce preparation time and expand the range of operational settings.

Beyond the ballast, transitioning the software framework from ROS to ROS 2 is another major milestone, enabling improved communication, modularity, and scalability for multi-robot deployments. We also plan to release an autonomy package that integrates acoustic networking through the BlueBuzz modem. Secondary improvements will address ease of assembly and maintainability, including redesigning thruster mounts for faster integration and replacement. Collectively, these developments will expand the capabilities of the MUR as an open-source research platform.

## Public repositories & links

See Table 6.

**Table 6:** Public repositories & links.

Public repository (Github)	github.com/scottmayberry/MUR
Public repository (Zenodo)	doi.org/10.5281/zenodo.14968240
Videos	YouTube; GitHub; Zenodo, Path: hardware/Assembly/assembly_videos/**

## MUR in literature

The MUR is part of μNet, a comprehensive open-source, open-architecture infrastructure designed to support research and education in underwater communication, networking, and cooperative autonomy. μNet integrates an indoor testbed [13] — featuring miniaturized robotic platforms like the MUR for controlled experiments — with a lake testbed [15] that employs commercial AUVs and boats for real-world field testing. Its shared aquatic infrastructure incorporates components such as an acoustic communication device enabling underwater connectivity [14], [16], as well as work in acoustic localization for precise positioning in confined environments [17]. By offering modular acoustic networking capabilities, μNet fosters collaboration among the robotics, signal processing, and networking communities while lowering the barriers to entry for underwater systems research.

## CRediT authorship contribution statement

**Scott Mayberry:** Writing – review & editing, Writing – original draft, Visualization, Validation, Software, Methodology, Investigation, Data curation, Conceptualization. **Jinzhi Cai:** Writing – review & editing, Validation, Software, Methodology, Investigation, Conceptualization. **Ruochu Yang:** Writing – review & editing, Validation, Software, Investigation. **Junkai Wang:** Writing – review & editing, Validation, Software, Investigation. **Fumin Zhang:** Writing – review & editing, Supervision, Project administration, Funding acquisition, Conceptualization.

## Ethics statements

This research did **not** involve human subjects, animal experiments, or any other ethical concerns requiring specific approval.

## Funding

This research is supported by 10.13039/100000001National Science Foundation, United States grants CNS-1828678 and CNS-2016582.

## Declaration of competing interest

The authors declare that they have no known competing financial interests or personal relationships that could have appeared to influence the work reported in this paper.
